# Epidemiology and Molecular Relationships of *Cryptosporidium* spp. in People, Primates, and Livestock from Western Uganda

**DOI:** 10.1371/journal.pntd.0001597

**Published:** 2012-04-10

**Authors:** Stephanie J. Salyer, Thomas R. Gillespie, Innocent B. Rwego, Colin A. Chapman, Tony L. Goldberg

**Affiliations:** 1 Department of Pathobiological Sciences, School of Veterinary Medicine, University of Wisconsin-Madison, Madison, Wisconsin, United States of America; 2 Department of Environmental Studies, Emory University, Atlanta, Georgia, United States of America; 3 Department of Biology, School of Biological Sciences, Makerere University, Kampala, Uganda; 4 Department of Anthropology and School of Environment, McGill University, Montreal, Canada; University of California San Diego School of Medicine, United States of America

## Abstract

**Background:**

*Cryptosporidium* is one of the most common parasitic diarrheal agents in the world and is a known zoonosis. We studied *Cryptosporidium* in people, livestock, and non-human primates in the region of Kibale National Park, Uganda. Land use change near the park has resulted in fragmented forest patches containing small, remnant populations of wild primates that interact intensively with local people and livestock. Our goal was to investigate risk factors for *Cryptosporidium* infection and to assess cross-species transmission using molecular methods.

**Methodology/Principal Findings:**

Diagnostic PCR revealed a prevalence of *Cryptosporidium* of 32.4% in humans, 11.1% in non-human primates, and 2.2% in livestock. In the case of humans, residence in one particular community was associated with increased risk of infection, as was fetching water from an open water source. Although 48.5% of infected people reported gastrointestinal symptoms, this frequency was not significantly different in people who tested negative (44.7%) for *Cryptosporidium*, nor was co-infection with *Giardia duodenalis* associated with increased reporting of gastrointestinal symptoms. Fecal consistency was no different in infected versus uninfected people or animals. DNA sequences of the *Cryptosporidium* oocyst wall protein gene placed all infections within a well-supported *C. parvum*/*C. hominis* clade. However, the only two sequences recovered from primates in the core of the park's protected area fell into a divergent sub-clade and were identical to published sequences from *C. parvum*, *C. hominis*, and C. *cuniculus*, suggesting the possibility of a separate sylvatic transmission cycle.

**Conclusions/Significance:**

*Cryptosporidium* may be transmitted frequently among species in western Uganda where people, livestock, and wildlife interact intensively as a result of anthropogenic changes to forests, but the parasite may undergo more host-specific transmission where such interactions do not occur. The parasite does not appear to have strong effects on human or animal health, perhaps because of persistent low-level shedding and immunity.

## Author Summary

*Cryptosporidium* is a common gastrointestinal parasite known for its zoonotic potential. We found *Cryptosporidium* in 32.4% of people, 11.1% of non-human primates, and 2.2% of livestock in the region of Kibale National Park, Uganda. In people, infection rates were higher in one community than elsewhere, and fetching water from an open water source increased the probability of infection. Phylogenetic analyses identified clusters of *Cryptosporidium* with mixed host origins in people, primates, and livestock outside the park; however, parasites from primates inside the park were genetically divergent, suggesting a separate sylvatic transmission cycle. Infection was not associated with clinical disease in people, even in the case of co-infection with the gastrointestinal parasite *Giardia duodenalis*. Parasites such as *Cryptosporidium* may be maintained through frequent cross-species transmission in tropical settings where people, livestock, and wildlife interact frequently, but the parasite may undergo more host-specific transmission where such interactions do not occur. Persistent low-level shedding and immunity may limit the clinical effects of infection in such settings.

## Introduction

*Cryptosporidium* is one of the most common diarrhea-causing parasitic genera in the world [1]. Its emergence as a significant human pathogen and its known zoonotic potential make it a threat to global public health [2]. *Cryptosporidium* infection is particularly problematic in AIDS patients [3], making it of special concern for populations with high rates of HIV infection. Unfortunately, relatively little is known about the ecology of *Cryptosporidium* in areas with high human-animal interaction that are also heavily burdened with HIV.

Uganda is a country of rich but declining natural resources, a high rate of human population growth, and a high HIV burden (estimated at approximately 6% of the population) [4]. In Uganda, studies of *Cryptosporidium* have focused on urban populations [5] or on risks to endangered mountain gorillas (*Gorilla gorilla beringei*) [6]–[8]. However, most of Uganda is rural, and mountain gorillas are rare and restricted to two small regions of the country; other wildlife species interact more commonly with people and domestic animals and may be more widespread sources and/or recipients of infection.

This study investigates *Cryptosporidium* infection in wild non-human primates (primates hereafter), people, and livestock in fragmented forest habitats near Kibale National Park, Uganda. Kibale contains one of the most biodiverse primate communities in Africa [9]. Kibale itself is well protected, but agricultural clearing has resulted in a series of forest fragments outside the park, containing small, remnant populations of primates [10]. In such locations, interactions among people, livestock, and primates occur frequently [11]. Previous research estimated a 14.3% prevalence of *Cryptosporidium* in red colobus monkeys in forest fragments near Kibale, but no infection in the same species in protected locations within the park [12]. The current study examines *Cryptosporidium* infection in local people, livestock, and primates and uses survey data to investigate how human demography, behavior, and interactions with animals affect infection risk. In addition, this study uses DNA sequence data to classify *Cryptosporidium* infections and to assess cross-species transmission. Our results shed new light on the ecology of *Cryptosporidium* in western Uganda and its effects on animal and human health.

## Materials and Methods

### Ethics Statement

All animal use followed the guidelines of the Weatherall Report on the use of non-human primates in research and was approved by the Uganda Wildlife Authority, the Uganda National Council for Science and Technology, and the University of Wisconsin Animal Care and Use Committee. Prior to data collection, all human subjects protocols were reviewed and approved by the Uganda National Council for Science and Technology as well as by the Institutional Review Boards of the University of Illinois and the University of Wisconsin, which approved oral consent due to low literacy rates. Oral informed consent was obtained by trained local field assistants and documented by witnessed notation on IRB-approved enrollment forms.

### Study site

The study took place in Kibale National Park, western Uganda (0°13′–0°41′N, 30°19′–30°32′E) and surrounding forest fragments. Kibale National Park consists primarily of moist semi-deciduous and evergreen forest, between approximately 1,100 and 1,600 m in elevation and is one of the most biodiverse locales for primates in the world [9]. The Kibale region is noted for its rapidly expanding human population and intense human-wildlife interaction and conflict [13]. This conflict is especially pronounced outside of the protected areas of Kibale, in remnant forest fragments that sustain small populations of certain primate species that come into frequent and often antagonistic contact with local people and their livestock [14].

For the present study, we focused on three such forest fragments: Bugembe, Kiko 1, and Rurama, and the households immediately surrounding them. These sites, which are described in detail elsewhere [15], range from 0.7 to 1.5 km^2^ and contain small populations of primates (between 4 and 60 individuals of up to three monkey species; see below).

### Sample collection and surveys

In May and June, 2007, the end of the rainy season, fecal samples were collected from two folivorous monkey species, the red colobus (*Procolobus rufomitratus*; n = 30) and the black-and-white colobus (*Colobus guereza*; n = 29), and one omnivorous monkey, the red-tailed guenon (*Cercopithecus ascanius*; n = 22). Collection sites included pristine/undisturbed forest within the park, as well as the highly disturbed Bugembe, Kiko 1, and Rurama forest fragments. Fecal samples and demographic information from primates were collected non-invasively during routine behavioral observations [15]. Fecal consistency was recorded as firm, soft, or loose for all samples collected. Exact ages of primates and livestock were not known, so these animals were classified into age categories (infant, juvenile, subadult, adult).

Samples were also collected from local human volunteers selected without respect to age, sex, or symptoms (n = 108; mean age of 23 years with an age range of 1.9 months to 75 years) and their livestock (cattle, *Bos taurus* and *B. indicus*, n = 25; goats, *Caprus hircus*, n = 57; and sheep, *Ovis aries*, n = 7; ages recorded as juvenile, subadult, or adult) in villages surrounding each forest fragment [15]. People and livestock in these villages use forest fragments intensively for such purposes as firewood collection and grazing, respectively, thus increasing their ecological overlap with primates [11]. Volunteers were instructed to place fecal samples into sterile cups, which were collected the following day. Fresh livestock feces were collected from the rectum using a sterile glove, or from the ground if the animal was observed to defecate, with care taken to avoid environmental contamination by sampling only fecal material that had not come into contact with the ground. Fecal samples were transported to a field laboratory as soon as possible after collection (within 6 hours). One milliliter of fecal material from each sample was homogenized with an equal volume of RNAlater nucleic acid stabilizing buffer (Ambion Inc., Austin, TX) and stored at −20°C in the field prior to transport to the United States.

Concurrent with human fecal sample collection, a survey was administered to each volunteer who provided a sample. The survey focused on demography, gastrointestinal symptoms (presence or absence within the previous 4 weeks), land use, medication, and interactions with animals during the four-week period prior to sample collection. To minimize response bias, surveys were administered by trained local field assistants in the local language (Rutooro). Data were manually recorded on paper forms, entered into spreadsheets in the computer program Microsoft Excel, and subsequently reviewed for accuracy. Associations between survey responses and infection status (see below) were evaluated using univariate Fisher's exact tests followed by multivariate regression in the computer program SPSS version 19.0 (SPSS Inc., Chicago IL). Specifically, univariate analyses of 25 different predictor variables were performed with no correction for multiple comparisons, and significant factors were then input into a multiple logistic regression with statistical significance set to 0.05 at all stages.

### Molecular methods

Total DNA was extracted from 250 µl of fecal and RNAlater suspension using the ZR Fecal DNA Kit (Zymo Research, Irvine CA) according to the manufacturer's protocol. As a quality control step for extraction efficiency and PCR inhibition, 4 µl of commercially available, non-interfering internal control DNA was added to each sample during the lysis step of the extraction, and extracted DNA was subjected to a primer-limited real-time quantitative PCR according to the recommendations of the manufacturer (PrimerDesign Ltd, Hampshire, UK). Real-time PCRs were run on a Bio-Rad CFX96 platform using 2× iQ Supermix master mix (Bio-Rad Laboratories, Hercules, CA). Only those samples yielding positive results for the internal control amplification were carried forward; samples that yielded negative results for the internal control amplification were re-extracted until positive internal control amplification was achieved.

To screen samples for *Cryptosporidium*, a nested PCR was performed on each sample, targeting a 311 bp segment of the *Cryptosporidium* oocyst wall protein (COWP) gene, based on published methods [16]. For all primary PCRs, 12.5 µl reactions were run with 0.625 U of FailSafe PCR Enzyme Mix (EpiCenter Biotechnologies, Madison, WI), 2 µl of template, 0.5 µM of primers Cry9 (5′-GGACTGAAATACAGGCATTATCTTG-3′) and Cry15 (5′-GTAGATAATGGAAGAGATTGTG-3′), and 6.25 µl of FailSafe buffer I per reaction. The thermal profile included an initial denaturation step for 5 min at 94°C, 30 cycles of denaturation at 94°C for 50 sec, annealing at 52°C for 30 sec, and extension at 72°C for 50 sec, with a final extension at 72°C for 10 min. Two microliters of each primary PCR product was then used in a secondary 25 µl nested reaction containing 0.5 U DyNAzyme EXT DNA polymerase (Finnzyme, Espoo, Finland), 10× DyNAzyme EXT buffer, 2.5 mM MgCl_2_, 0.2 mM dNTP mix, 0.5 µM each of primers CnestF (5′-TGTGTTCAATCAGACACAGC-3′) and CnestR (5′-TCTGTATATCCTGGTGGGC-3′). The same thermal profile was used for the secondary PCR, except that the annealing temperature was 60°C.

Secondary PCR products were run on 2% GenePure LE Quick Dissolve agarose gels (ISC BioExpress, Kaysville, UT) containing 20 µg/100 ml ethidium bromide in 1× TAE buffer at 80 volts for approximately 1 h. Bands of the predicted size were visualized using a UV light source, cut from the gel, and then extracted using the Zymoclean Gel DNA Recovery Kit (Zymo Research, Irvine, CA). Extracted DNA was analyzed by a NanoDrop 1000 spectrophotometer (Thermo Scientific, Wilmington, DE) to determine DNA concentration. Gel-purified DNA was then sequenced in both directions using internal primers CnestF and CnestR at the University of Wisconsin Biotechnology Center (Madison, WI) on ABI 3730xl DNA Analyzers (Applied Biosystems, Carlsbad CA).

Sequences were used for general assignment of infections to taxonomic groups. Sequences were edited using Sequencher v4.9 (Gene Codes Corporation, Ann Arbor MI). Edited sequences were then aligned using the computer program ClustalX [17] with minor manual adjustment. BLAST searches were conducted with edited sequences to determine the similarity of *Cryptosporidium* presenting Ugandan samples to published sequences. To determine the taxonomic positions of newly generated COWP sequences relative to representative published sequences, phylogenetic trees were constructed using the neighbor joining method [18] with maximum composite likelihood distance correction in the computer program MEGA, version 5.0 [19], with robustness of groupings assessed using 1000 bootstrap replicates of the data [20]. Molecular data on the presence of *Giardia duodenalis* in these same samples determined by real-time PCR for the small subunit RNA gene [21] were already available from a previous study [22]. All sequences generated during this study were deposited in GenBank (accession numbers JF342450–JF342495).

## Results

Using PCR, we detected *Cryptosporidium* in 46 (16.4%) of 280 fecal samples (Table 1). The prevalence of *Cryptosporidium* was higher in humans (32.4%) than in either non-human primates (11.1%) or livestock (2.2%). The prevalence of *Cryptosporidium* in humans was markedly higher in the Bugembe community (59.6%) than in either Kiko 1 or Rurama communities (22.2% and 20.2%, respectively; Table 1), and this difference was statistically significant (Odds ratio = 5.48; 95% confidence interval: 2.239–13.419; Fisher's exact P = 0.0002; Table 2). A correspondingly higher prevalence of *Cryptosporidium* was detected in both livestock and primates in Bugembe (Table 1), although neither trend was statistically significant (Fisher's exact P = 0.059 and 0.349, respectively; Table 2).

**Table 1:** Prevalence of *Cryptosporidium* infection by species and location determined by diagnostic PCR.

Host	Location^a^							
	Kibale National Park		Kiko 1		Bugembe		Rurama	
	+/Total	Prevalence (95% CI)	+/Total	Prevalence (95% CI)	+/Total	Prevalence (95% CI)	+/Total	Prevalence (95% CI)
Human	n/a		8/36	0.22 (0.12–0.38)	19/32	0.59 (0.42–0.75)	8/40	0.20 (0.10–0.35)
Cattle	n/a		0/13	0 (0.00–0.27)	0/6	0 (0.00–0.44)	0/6	0 (0.00–0.44)
Goats	n/a		0/20	0 (0.00–0.19)	2/21	0.1 (0.02–0.30)	0/17	0 (0.00–0.22)
Sheep	n/a		0/4	0 (0.00–0.55)	n/a		0/4	0 (0.00–0.55)
Red colobus	2/8	0.25 (0.06–0.60)	n/a		3/12	0.25 (0.08–0.54)	3/10	0.30 (0.10–0.61)
Black-and-white colobus	0/10	0 (0.00–0.32)	n/a		1/9	0.11 (0.00–0.46)	0/10	0 (0.00–0.32)
Red-tailed guenon	0/10	0 (0.00–0.32)	n/a		0/2	0	0/10	0 (0.00–0.32)

a People and livestock do not inhabit Kibale National Park, and non-human primates have been extirpated from Kiko 1 forest fragment, so prevalence data are not available for these species/locations (indicated by “n/a”).

**Table 2:** Risk factors for infection with *Cryptosporidium* spp. in people living near Kibale National Park.

			95% CI		
Variable^a^	n^b^	OR	Lower	Upper	P^c^
Age (≤15 years)	107	0.801	0.355	1.809	0.678
Sex (female vs. male)	107	1.484	0.659	3.341	0.411
**Location (Bugembe vs. Kiko 1 or Rurama)**	108	5.48	2.239	13.419	0.0002
Location (Rurama vs. Kiko 1 or Bugembe)	108	.380	.152	.947	.054
Location (Kiko 1 vs. Bugembe or Rurama)	108	.476	.190	1.194	.130
Worked in agricultural fields	90	0.878	0.341	2.26	0.812
Worked in the forest (e.g. collected firewood)	90	1.81	0.6895	4.728	0.3295
**Fetched water from an open water source**	90	3.75	1.449	9.703	0.008
Fetched water from a closed well	90	1.004	0.422	2.388	1.0
Tended livestock	90	0.61	0.2417	1.545	0.4195
Lived in household with another positive person	108	2.1	0.838	5.266	0.130
Lived in household with positive livestock	108	6.75	0.676	67.404	0.099
Experienced gastrointestinal symptoms	86	0.906	0.380	2.162	1.0
Used traditional or commercial medicines	90	1.082	0.454	2.578	1.0
Co-infection with *Giardia duodenalis*	108	0.801	0.350	1.832	0.678

a Variables in bold were retained in a final multiple logistic regression model (see text).

b Numbers of observations differ due to incomplete reporting of information on some surveys.

c Two-tailed P values were calculated using Fisher's exact tests.

Data from survey responses (83.5% of people responding) were used to identify risk factors for human *Cryptosporidium* infection related to land use, demography, and behavior (Table 2). Fetching water from an open source (e.g. a pond or stream) was significantly associated with infection status (Odds ratio = 3.75; 95% confidence interval: 1.449–9.703; Fisher's exact P = 0.008). Other factors related to behavior were not statistically associated with infection, including working in fields, working in the forest, fetching water from a protected well, or tending livestock (Table 2). We found no association between infection with *Cryptosporidium* and the reporting of any gastrointestinal symptom, age, taking of medicines, or being co-infected with *Giardia duodenalis* (Table 2). Infection with *Cryptosporidium* was not significantly associated with reporting of gastrointestinal symptoms in humans, nor were any such associations found when volunteers were stratified by age (decades). Fecal samples from people were all normal in consistency.

To control for potential confounding, we used multiple logistic regression with various strategies of model selection (e.g. forward addition, backward stepwise elimination). Regardless of the strategy used, the same two variables were retained in the final model in all cases: fetching water from an open source (adjusted odds ratio = 2.965; 95% confidence interval: 1.100–7.994; P = 0.032) and residence in Bugembe (adjusted odds ratio = 3.404; 95% confidence interval: 1.314–8.818; P = 0.012). Although univariate analyses suggested a positive association between infection and residency in a household with at least one other *Cryptosporidium* positive person or with at least one positive cattle, goat, or sheep, these trends were not significant when multiple logistic regression was performed (Table 2).

For primates, the infection rate was 11.1% overall (Table 1). Contrary to our previous results [12], primates in forest fragments were not infected at a statistically significantly higher rate than primates in protected areas of the park (Odds ratio = 0.53; 95% confidence interval: 0.10–2.72; Fisher's exact P = 0.358). Infection rates in domestic animals were 2.2% overall, with no significant differences based on species or location (Table 1). Fecal consistency was normal for all animals sampled and thus not statistically different in infected versus uninfected primates or livestock.

**Figure 1:**
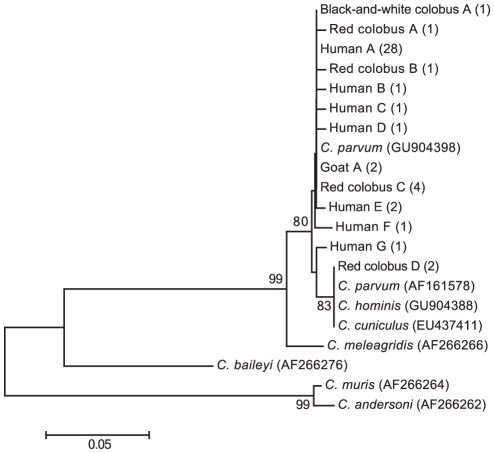
Dendrogram of *Cryptosporidium* from people, primates, and livestock in the region of Kibale National Park, Uganda. The tree was constructed from partial nucleotide sequences of the outer wall protein gene using the neighbor-joining method [18] and a maximum composite likelihood distance correction, implemented with the computer program MEGA, version 5.0 [19]. Numbers indicate bootstrap values (%), estimated from 1,000 resamplings of the sequence data [20]; only bootstrap values ≥50% are shown. Taxon names indicate the host species from which each sequence was recovered, followed by an arbitrary letter designation and numbers of individuals from which the sequence was recovered in parentheses. Reference sequences are shown with GenBank accession numbers in parentheses. GenBank accession numbers of newly generated sequences are JF342450–JF342495. The scale bar indicates nucleotide substitutions per site.

Phylogenetic analysis of a 231-bp alignment of the COWP gene using newly generated and representative published sequences confirmed that all *Cryptosporidium* sequences generated from Kibale clustered within a clade containing known variants of *C. parvum* and *C. hominis* (Figure 1). Within this clade, some sequences recovered from black-and-white colobus, red colobus, and people were identical. Intriguingly, the only two sequences recovered from red colobus in the core protected area of Kibale National Park clustered distinctly from sequences recovered from people, primates, and livestock in communities and forest fragments outside of the park. These two red colobus-derived sequences (Red colobus D in Figure 1) were identical to each other and to published sequences attributed to *C. parvum*, *C. hominis*, and *C. cuniculus*, which formed a divergent sub-clade.

## Discussion

Our results demonstrate that the prevalence of *Cryptosporidium* in people in rural western Uganda is high, with up to approximately two thirds of people infected in some locations. These results are similar to those of other studies that have documented high rates of *Cryptosporidium* infection in impoverished rural populations with limited access to health care, (e.g. [23]–[25]). Our prevalence estimates are considerably higher than those reported in people living outside of Bwindi National Park, Uganda [8], or in children hospitalized with diarrhea in Kampala, Uganda [5]. The greater prevalence documented in our study may reflect real ecological differences among the study areas, possibly with respect to hydrology or seasonality, since our study was performed at the end of the rainy season. Alternatively, this difference could reflect the higher sensitivity of our PCR-based detection method as compared with the microscopic methods used in these other studies. Although the prevalence estimate we report here for red colobus in forest fragments (27.3%) was comparable to what we previously estimated (20.0%) using immunofluorescent microscopy [12], we did not detect *Cryptosporidium* infection at higher rates in red colobus in forest fragments than in protected areas of the park, as in our previous study [12].

People from the Bugembe community were infected with *Cryptosporidium* at a rate approximately three times higher than that of people from the Rurama or Kiko 1 communities, despite the roughly similar demographic composition of these communities and their close proximity to each other (within 7 km). Similarly, livestock and primates in Bugembe were also infected with *Cryptosporidium* at comparatively higher rates, although this trend was not statistically significant. The reasons for these trends are not clear, but they nevertheless underscore that the prevalence of infection with *Cryptosporidium* can vary considerably over fine spatial scales. Given that *Cryptosporidium* is a waterborne pathogen [26], positive association between infection and fetching water from an open source was not surprising. We speculate that differences in *Cryptosporidium* infection rates among communities may reflect variation in water quality.

We found no measurable association between infection of people with *Cryptosporidium* and the reporting of gastrointestinal symptoms, even when co-infection with *G. duodenalis* and age were also considered. This may have been a consequence of our small sample sizes, but it is concordant with the published literature. For example, a two-year prospective cohort study of 207 Peruvian children revealed that 63% of children developing their first *Cryptosporidium* infection were asymptomatic (*i.e.* had no diarrhea [27]); however these children still showed decreased weight gain compared to those not infected. While specific measurements such as body mass index were not collected in our study to investigate factors such as malnutrition, such data could be included in future studies. In addition, persistent asymptomatic oocyst excretion can extend beyond clinical illness [28]–[30], making chronic post-symptomatic shedding also a likely cause for the observed lack of clinical effects in our study. Finally, other studies indicate that asymptomatic cryptosporidiosis may be common in immunocompromised individuals [31]–[34]. The overall high prevalence of *Cryptosporidium* infection documented in our study could therefore reflect poor immune status, perhaps due to poor nutrition or HIV/AIDS infection, which is very common in this region [35]. Although we did not have access to data on the HIV infection status of volunteers included in this study, we propose that future studies of the interactions among hygiene, nutrition, animal contact, and HIV infection in this region might prove informative.

We did not find evidence of any effects of *Cryptosporidium* infection on the consistency of fecal samples in primates or livestock, because all animal samples had normal fecal consistency. Nevertheless, we cannot discount the possibility that *Cryptosporidium* is impacting the health of these animals, which could have negative conservation implications. The Ugandan red colobus monkey, for example, is endangered and may be declining due to a combination of nutritional stress and parasitism [36]. Increasing human encroachment into primate habitats and consequent human-primate conflict will likely intensify such trends [11].

Our molecular analyses showed that people and livestock in Kibale are infected with *Cryptosporidium* variants that lie within a well-supported *C. parvum*/*C. hominis* clade, although COWP gene sequences did not distinguish between these two species. The majority of sequences from the Kibale region clustered closely with known *C. parvum*, with several sequences being identical regardless of whether they were recovered from people, primates, or livestock. This observation suggests that cross-species transmission of *Cryptosporidium* may be common in the region, which would be consistent with our previous studies demonstrating frequent cross-species transmission of *Escherichia coli* and *G. duodenalis* [15], [22], [37]. Given the similar ecologies and modes of transmission of these microbes, we interpret this finding as evidence of general “pathogen pollution,” reflecting increased risk of infection from environmental sources (water, vegetation, soil) in areas of intense human/animal overlap.

Our molecular classifications of *Cryptosporidium* infections should be viewed as preliminary, given our single-locus approach. Although COWP has been widely used as a taxonomic marker, recent methodological advances promise to increase the resolution of molecular data for studies of *Cryptosporidium* taxonomy and epidemiology. For example, multilocus typing approaches are advancing rapidly [38], including for non-human-associated taxa such as *C. muris* and *C. andersoni* [39]. The sequencing of the *C. parvum* and *C. hominis* genomes and the advent of novel “deep” sequencing technologies should further enhance such efforts [40]. In the absence of an efficient *in vitro* culture system for *Cryptosporidium*, however, application of molecular methods to epidemiological studies will still require initial PCR of *Cryptosporidium* DNA from complex clinical samples such as feces, which has proven technically challenging in many studies, including ours.

Despite these caveats, we note that the only two *Cryptosporidium*-positive red colobus monkeys in the core protected area of Kibale National Park yielded COWP sequences that were divergent from those recovered from hosts outside of the park. This finding raises the possibility of separate *Cryptosporidium* transmission cycles inside and outside of the park. Sylvatic *Cryptosporidium* transmission cycles are poorly understood, and we are unaware of any studies of the effects of *Cryptosporidium* infection on the health or fitness of primates in their natural environments. We therefore view the results of this study as indicating future avenues for research into the transmission of *Cryptosporidium* in humans and wildlife, focused not only where these species interact in anthropogenically disturbed habitats, but also in protected areas that can serve as baselines for comparison.
